# Determination of Material and Fracture Properties of a Case-Hardened Planet Gear and Its Homogenisation Method to Obtain the Damage Mechanism Caused by Fragment Ingestion

**DOI:** 10.3390/ma17020366

**Published:** 2024-01-11

**Authors:** Julia Jeßberger, Christian Fischer, Stephan Rinderknecht

**Affiliations:** Mechatronic Systems in Mechanical Engineering, Technical University of Darmstadt, Otto-Berndt-Straße 2, 64287 Darmstadt, Germanyrinderknecht@ims.tu-darmstadt.de (S.R.)

**Keywords:** Johnson-Cook (J-C) model, plastically graded materials (PGM), homogenisation, fracture

## Abstract

Before a new type of engine is introduced into civil aviation, it must comply with various safety regulations. These regulations include the analysis of secondary damage caused by the re-ingestion of a tooth fragment. The purpose is to prevent crack propagation through the gear rim, which would lead to catastrophic failure. In this context, identification of the initial crack location is crucial to determine the crack propagation path. Therefore, this paper presents a technique to determine and validate a constitutive material model and fracture locus for case-hardened spur gears. As the modelling of the surface-hardened layer is computationally intensive, it is necessary to homogenise the model. This paper comprehensively reviews and discusses the associated effects and errors. To determine the plastic behaviour of the case-hardened external gear (30CrNiMo8) and the nitrided internal gear (35CrAlNi7-10), the widely acknowledged Johnson–Cook material model is implemented using compression and Vickers indenter tests to define the necessary parameters. The fracture locus implementation is also based on the Johnson–Cook method and an axial shift of the fracture locus based on the hardness profile of the spur gears is determined by quasi-static pulsator tests. For validation, a project-specific gearbox test rig is used, enabling consistent ingestion of defined fragments. In addition, to check the likelihood of a tooth flank crack and to validate the results, a simplified ingestion experiment is performed.

## 1. Introduction

For the market launch of a new engine type with a gearbox, certification is required. Therefore the Certification Specifications for Engines follow the rules of the European Aviation Safety Agency (EASA CS-E). The aim is to detect potentially hazardous effects for the Power Gear Box (PGB) like the release of high energy debris (HED) and PGB seizure leading to a locked Fan. For the certification, a certain likelihood of potentially hazardous engine effects needs to be ensured. It is crucial to distinguish between benign and catastrophic cracks. The former is defined as propagation through the gear teeth, while the latter is defined as propagation through the gear rim. The probability of catastrophic failure must be reduced to a minimum. The likelihood of catastrophic failure must be reduced to a minimum. In contrast, benign failure is permitted. So far, there is no literature available on the repetitive ingestion of a specified metal fragment into the gearing of a gearbox. However, two studies were found on the ingestion of an undefined metal fragment without any metrological evaluation [1,2]. In both examinations, nevertheless, there was only plastic deformation of the fragment, but no fracture of the gears. Therefore, this study examines the failure mechanism resulting from the re-ingestion of a metal fragment that is both tooth-sized and trough-hardened, with precise accuracy, into a quasi-static rotating planetary gearbox. Since experiments are costly, time-consuming and difficult to access with measurement equipment, it is necessary to implement and validate a numerical model additionally. Due to the high computational effort in the numerical approach that considers the hardened surface of the gears, homogenisation is necessary for this study. This paper shall not employ any black-box models. Instead, a grey box model will be used with a suitable strategy for determining the constitutive material properties, including the strain hardening factor, elastic modulus and yield stress, as well as the material constants of the fracture locus for both the surface-hardened and the homogenised model.

The indenter technique is a well-established method for determining the constitutive material properties of surface layers, as these tests are easy to repeat and can be carried out on small samples. Assumptions regarding the behaviour of Young’s modulus and strain hardening factor within the surface layer vary, and it can be concluded that the behaviour is material-dependent. Nayebi et al. [3] predicted the decreasing hardness profile of nitrided steels using instrumented ball indentation and finite element analysis based on the effective evolution of hardness. The method has been evaluated using four different steels with constant strain hardening factor and Young’s modulus throughout the material. Cao et al. [4] extended the method for sharp indentation to spherical indentation and identified the representative strain as a function of indentation depth and indentation radius. He determined the plastic properties, with varying strain hardening factors, based on an inverse approach. Moussa et al. [5] used spherical indentations to investigate the material properties of surface-hardened materials. It is argued that the assumption of no variation of the strain hardening exponent is incorrect since results indicate a decrease in the surface layer compared to the core material. Branch et al. [6] investigate the stress–strain behaviour of case-hardened steels as a function of depth using experimental and numerical methods. He utilised the variation in Micro-Vickers hardness with depth for both virgin and pre-deformed plastically graded materials. It is shown that this specific material has a linear variation in yield strength but a constant strain hardening exponent. To determine the strength coefficient as well as the strain hardening factor he used the compression test. The disadvantage of the Vickers indenter test is the necessity of reevaluation in order to convert the measured hardness to determine the yield stress. In order to reevaluate the hardness into yield stress, Tabor established one of the most popular analytical equations [7]. He used the correlation $C=\frac{H}{\sigma_{y}}=3$ for perfectly plastic engineering materials. Thereby, H represents the Vickers hardness and $\sigma_{y}$ the yield stress. For materials with strain hardening, the yield stress $\sigma_{y}$ is replaced by a representative stress $\sigma_{r}$ at a representative plastic strain $\varepsilon_{r}$. This concept was also first introduced by Tabor [8]. In the case of a conical indenter, the following relationship can be established between the constraint factor $C_{F}$, the hardness H and the flow stress $\sigma_{r}$ at a representative plastic strain $\varepsilon_{r}$ [7]:

(1) $$C_{F}=\frac{H}{\sigma_{r}\left(\varepsilon_{r}\right)}$$

So far, the literature has not agreed on a single value for the representative strain [8,9,10,11,12,13,14,15]. Tabor [8] performed experiments on two materials, mild steel and pure copper, and defined a representative strain $\varepsilon_{r}=0.08$. This value is determined so that the ratio between the mean pressure H and the corresponding representative stress $\sigma_{r}\left(\varepsilon_{r}\right)$ is equal to $C_{F}=3.3$. Another well-known numerical approach was made by Mata et al. [9] based on a variety of steels. The constraint factor was found to be $C_{F}=2.7$, and the representative strain was obtained to be $\varepsilon_{r}=0.1$.

In addition to the plastic deformation, a crack in the planetary gear is expected as part of the investigation. It is still necessary to determine the conditions that initiate a crack in the tooth flank or root.

The tooth root failure is one of the typical failure phenomena known in the literature, which can occur with overload. The crack initiation point is located on the surface of the tooth root. The strength of the tooth root can be estimated based on ISO6336/3 or American Gear Manufacturers Association (AGMA) or according to contact analysis based on higher-order deformation theory, which is known to be more precise [16]. Some researchers use quasi-static pulsator tests as an analogue test to document the cracking behaviour of spur gears [16]. Here, the maximum tooth root strength can be determined by continuously increasing the force until an initial crack occurs.

Less frequently, flank breakage or tip breakage occurs at overloading [17]. The first calculation methods to determine the local material strength refer to the shear stress intensity hypothesis, whereas Hertter [18] has derived a practice-oriented model whose calculation is directly dependent on the existing hardness profile and can thus be directly determined by a hardness profile depending on the distance from the surface. The dependency of the maximal shear strength $\tau_{zul}$ to the hardness H of the material is shown in Equation (2). Witzig [19] has calibrated the value $K_{\tau ,zul}=0.4$ based on results of experimental gear running tests. There are only a few publications regarding an analogue test that examines tooth flank fracture. Konowalczyk et al. [20] develops such a concept. He uses separated tooth segments which are loaded by two actuators on the double pulsator test rig.

In the case of helical gears, facing edge tooth flank fractures can occur. As a result of unfavourable deformation and stress behaviour in the area of the tooth face, local overloading of the material occurs, leading to the initiation of cracks near the surface of the active tooth flank [21].

(2) $$\tau_{zul}\left(y\right)=K_{\tau ,zul}\;\cdot \;H\left(y\right)$$

Regarding failure mechanisms of ductile material in general the relationship between equivalent strain at fracture and stress triaxiality $\eta =\frac{\sigma_{m}}{\sigma_{\mathrm{eq}}}$ (normalised hydrostatic pressure) has been investigated for many metallic alloys by various authors [22,23,24]. $\sigma_{m}$ thereby defines the hydrostatic stress and $\sigma_{\mathrm{eq}}$ the equivalent stress. A decreasing strain with increasing stress triaxiality was found for high-stress triaxiality ($\eta \geq \frac{1}{3}$). One of the best-known failure models with monotonic behaviour is that of Johnson–Cook [23], which is an extended version of the model from Rice and Tracey [25] and is dependent on temperature and strain rate. The analytical correlation between fracture strain $\varepsilon_{f}$ and stress triaxiality η by neglecting temperature and strain rate-dependent effects is

(3) $$\varepsilon_{f}=D_{1}+D_{2}\;\cdot \;exp\left(D_{3}\;\cdot \;\eta \right)$$

and defined by the material constants $D_{1},D_{2}$, and $D_{3}$.

**Figure 1:**
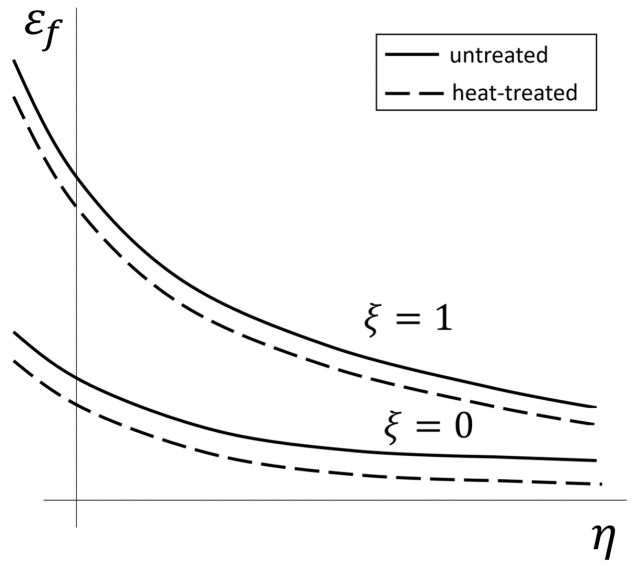
Upper and lower bound of the fracture locus for untreated and heat-treated materials.

Xue et al. [26] confirmed that a plane and an axisymmetric stress state lead to different parameters of the failure model, with the axisymmetric being the upper bound (ξ=1) and the plane strain state being the lower bound (ξ=0) of the failure strain. The parameter ξ refers to the stress state. The two states ξ=1 and ξ=0 depend on different parameters of the failure model (Figure 1).

In addition to the dependence on the stress state, Ghazali et al. [27] report a dependence of the heat treatment on the failure parameters. Regarding Ghazali, heat-treated specimens have a downwards-shifted fracture locus (Figure 1). As surface-hardened gears are used in the tests, the failure behaviour deviates from the fracture locus that can be determined from unhardened or through-hardened compression, shear and tensile tests. It is therefore necessary to find a strategy that allows the determination of a curve for such components. This paper aims to determine the fracture locus based on analogue tests for gear fracture under overload. It is also necessary to ensure that the initial crack location determined for the homogenised model does not deviate from the complex model with the surface layer taken into account.

## 2. Experimental Approach

The experiments have been categorised into two application purposes. The first part comprises tests conducted to determine and validate the constitutive material model. These include compression tests, Vickers indenter tests, and quasi-static ingestion tests into the scaled gear. The second part involves quasi-static pulsator tests and Fragment-Ingestion Tests (FIT) to determine and validate the fracture locus. The basic geometry of the cylindrical involute gear samples [28] used in this study is listed in Table 1. The planetary gearbox consists of three planets.

### 2.1. Experimental Approach to Determine Constitutive Material Model

#### 2.1.1. Compression Test

**Figure 2:**
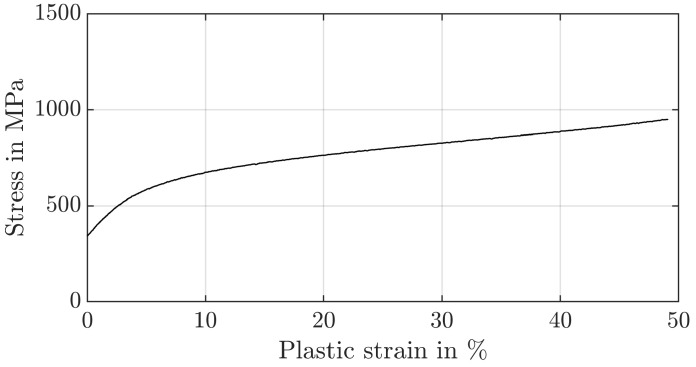
Stress–strain curve of the planet material (30CrNiMo8).

**Figure 3:**
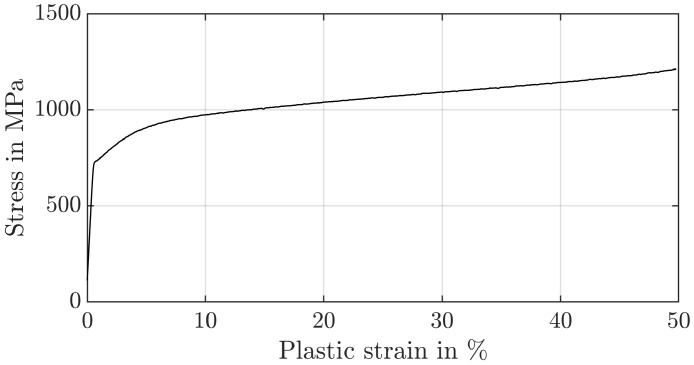
Stress–strain curve of the ring material (34CrAlNi7-10).

To identify the strain hardening factor *n* and to validate the re-evaluation strategy for the hardness with the yield stress $\sigma_{y}$ of the core material of the nitrided ring gear (34CrAlNi7-10) and the case-hardened sun and planet gears (30CrNiMo8), compression tests are carried out from the untreated core material (Figure 2 and Figure 3) according to DIN 50106 [29] on the universal testing machine RM1000 (MS-009) (Schenck, Darmstadt, Germany). For this purpose, the samples were positioned centrally between two plane-parallel plates and the deformation of the sample was recorded with two incremental displacement transducers offset by 180° (mean value). To minimise friction in the contact area between the specimen and the plunger, the faces of the compression specimens were lubricated with Molykote BR2plus. The stiffness of the test set-up was recorded at the beginning and taken into account later on. A total plastic strain of 50% was applied. Samples with a diameter of 10 mm are used. The height is varied (10 mm, 15 mm, 20 mm) to account for the effect of the diameter/height ratio. For the total plastic compression of the specimens, a force of 150 kN is required for the planet/sun gear and a force of 190 kN for the ring gear. The influence of the specimen height is not negligible. For specimens with a height of 20 mm, asymmetric deformations occur during the test. Since the test results for a height of 10 mm and 15 mm were in agreement and the height of the gear tooth is around 10 mm, these results are therefore used. Fracture is not observed in any of the tests. The yield stress can be estimated based on the 0.2% rule. The experimentally determined yield stress of the externally toothed gears is calculated to be $\sigma_{y}=322\;MPa$ and for the ring gear $\sigma_{y}=722\;MPa$.

#### 2.1.2. Vickers Indenter Test

**Figure 4:**
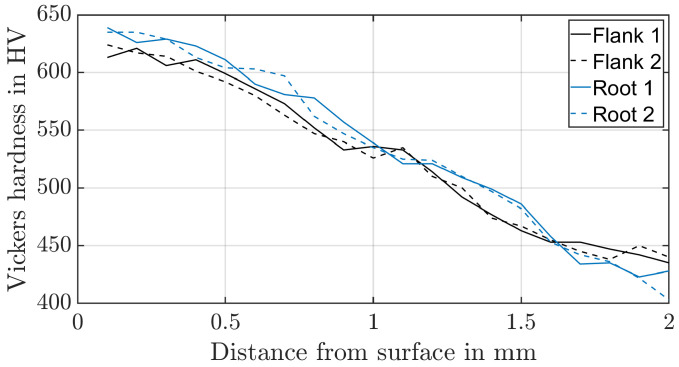
Hardness profile of the planet material (30CrNiMo8).

**Figure 5:**
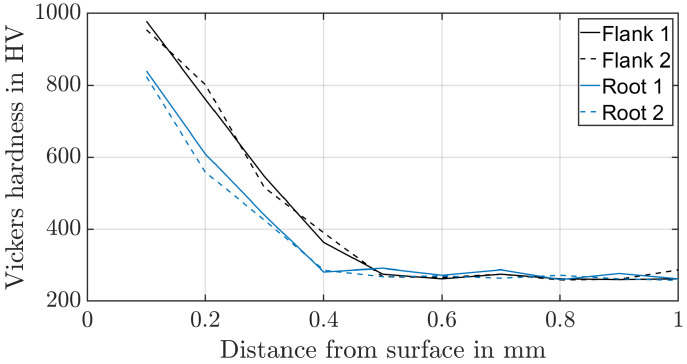
Hardness profile of the ring material (34CrAlNi7-10).

To identify the material properties of the surface layers, Vickers indenter tests based on DIN EN 18203:2022-07 [30] are carried out. A Vickers hardness of HV1 is used for the planet and sun, and a Vickers hardness of HV0.5 is used for the ring gear (Figure 4 and Figure 5). Two hardness profiles, each on different teeth, were investigated both in the tooth root and in the upper area of the tooth flank. The stress–strain curve obtained from the compression test, and the Vickers hardness of the core are used to validate the applicability of the reevaluation strategy based on the Equation (1) and the constraint factor and the representative strain of Mata et al. [9]. The relative error between the converted Vickers hardness and the measured yield stress of the compression test is about 1% for the ring gear material. The core area of the externally toothed gears was influenced by case hardening as well. For this reason, no error can be calculated.

#### 2.1.3. Quasi-Static Ingestion of a Fragment into a Scaled Gearbox

**Figure 6:**
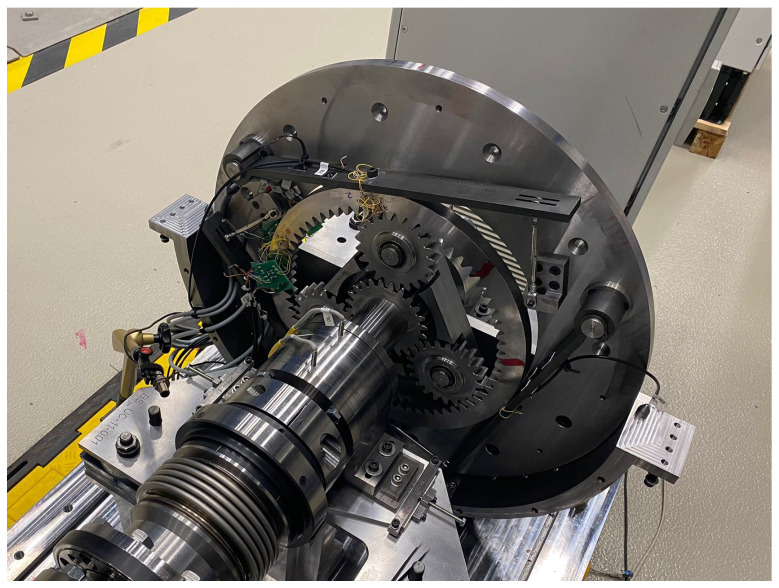
Illustration of the planetary gearbox used on the test rig.

**Figure 7:**
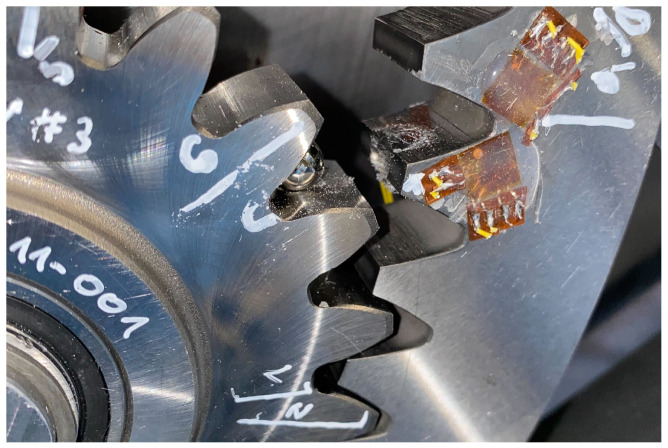
Plastic deformation after ingestion of a spherical fragment with a diameter of *D* = 8 mm.

**Figure 8:**
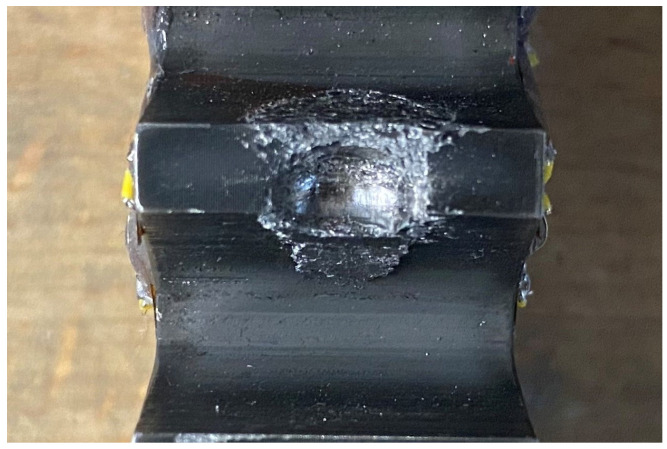
Plastic deformation after ingestion of a spherical fragment with a diameter of *D* = 8 mm—top view.

To detect potentially hazardous effects, tooth-sized fragments were ingested into the toothing and the failure mechanisms were detected. Since tests on the PGB are on the one hand extremely expensive and time-consuming and on the other hand difficult to access for measurement equipment and for ingesting the fragment repetitively, a single-stage, involute-toothed, high-performance gearbox is scaled down to a two-shaft configuration with a fixed thin-walled ring gear (Figure 6). To enable a highly dynamic ingestion planned for the future, spur gears are used. The fragment is ingested between the ring and the planet gear, sitting in the tooth gap of the planet gear. Various test scenarios were investigated as part of the quasi-static test series (Table 2). The term “25” means, the fragment is located 25% into the gear width measured from the front of the gear. Similarly, for the “75”, the fragment is located 75% deep into the toothing. Within the position “50”, the fragment is located in the centre of the tooth width. The ingestion of the fragment between the ring gear and the planet gear leads to noticeable plastic deformation but no visual crack for the spherical fragment and the smallest cylindrical fragment. Larger cylindrical fragments could not be completely rolled over quasi-statically due to test rig hardware-wise torque limitation. Experimental results of the quasi-static test rig with the scaled gearbox can directly validate the material behaviour up to the strain hardening area. Figure 7 and Figure 8 show the plastic deformation caused to the ring gear when a sphere with a diameter of 8 mm is ingested.

### 2.2. Numerical Approach to Determine Failure Model

**Figure 9:**
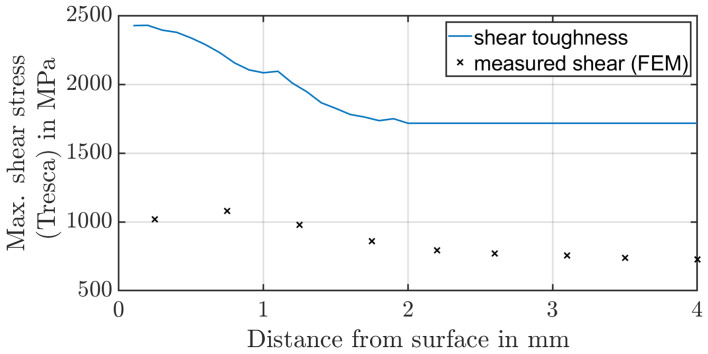
Comparing shear toughness calculated based on Hertter [18,19] with the measured shear stress.

Since failure of the gear is expected at the flank and root, the fracture locus of the surface-hardened gear is to be determined by analogous tests for overload fracture of gears. The quasi-static pulsator test is a suitable analogue test for root fracture. Using the simplified analytical calculation rule of Equation (2) and the calibrated value of Witzig [19], it can be deduced that even without considering the residual compressive stresses, the induced loads should not lead to a crack characteristic comparable to flank fracture (Figure 9). Since there is no analytical calculation rule or analogous test for facing edge tooth flank fracture that can be utilised for such a fragment-tooth contact, the Fragment-Ingestion Test (FIT) is used to determine the risk of fracture at the flank. The experiments use real gears with residual stresses that affect the strength of the material. Since the comparison between the experiment and the numerical simulation is based on the displacement of the fragment and the force required, and thus the same deformation state is present, it is assumed that this effect is indirectly considered by a shift caused by the constant residual stresses within the strains and stresses of the numerical model compared to the experiment. Since no effect on the initial crack position could be detected, as will be shown later in the paper, it is assumed that a displacement of the occurring stresses due to residual stresses will not affect the initial crack position.

#### 2.2.1. Quasi-Static Pulsator Test

**Figure 10:**
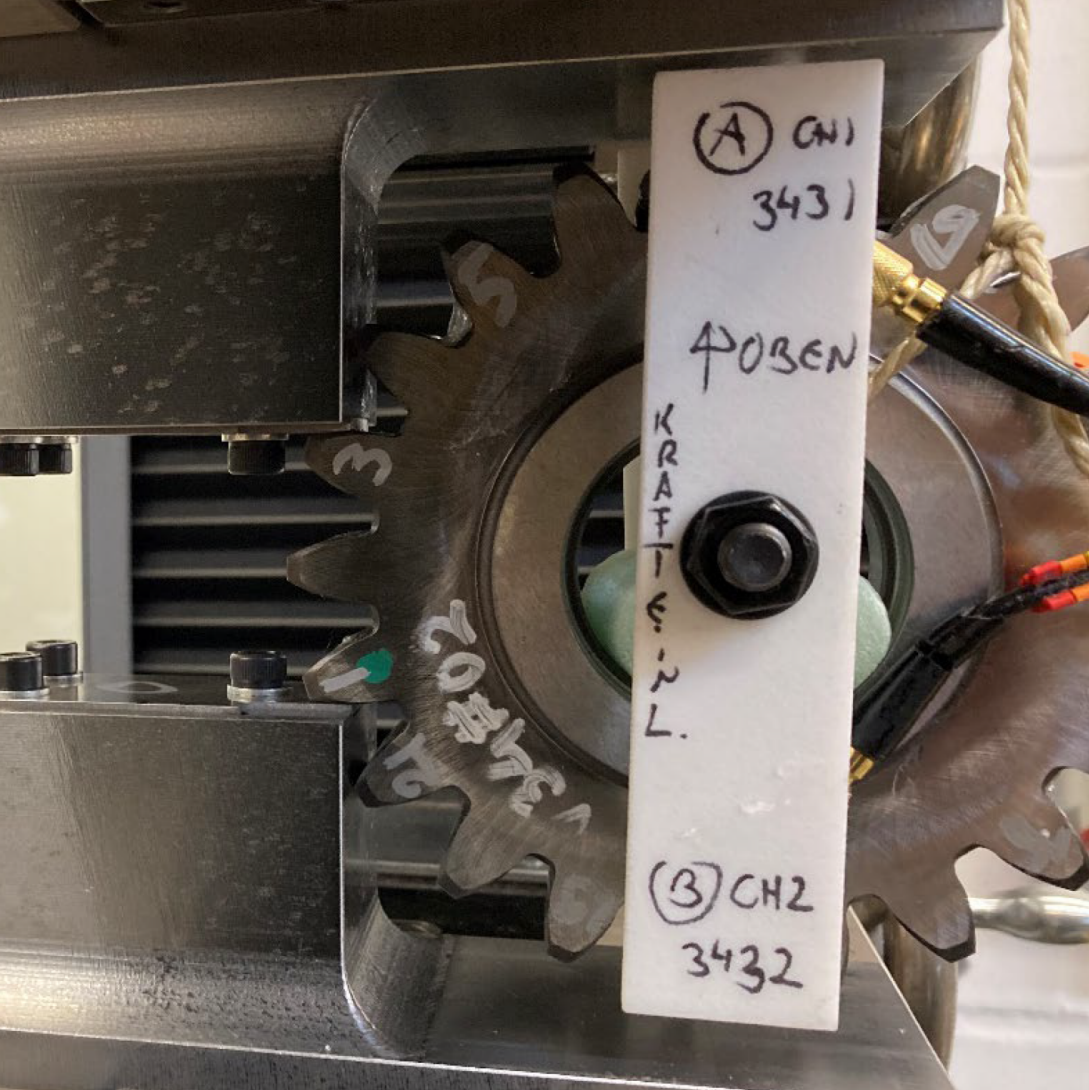
Experimental setup of the pulsator test.

**Figure 11:**
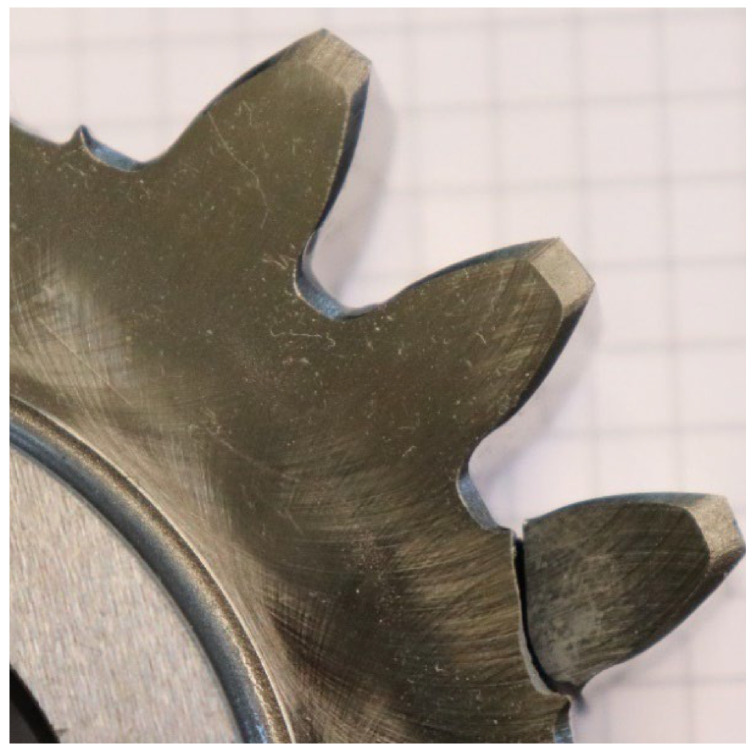
Crack propagation within the pulsator test.

To determine the fracture criterion of the tooth root, a quasi-static pulsator test is conducted on the planetary spur gear. As the clamped teeth are compressed, the force on them is increased until a crack appears at the root. The AE system detects the initial crack by measuring the transient elastic waves. These waves result from a sudden redistribution of stress in the component, such as that caused by a crack or crack growth. Figure 10 shows the test setup whereas Figure 11 shows the resulting crack. The results of the tests are given in Table 3 and indicate a considerable variation in the measured force, ranging from approximately 87 kN to 111 kN.

#### 2.2.2. Fragment-Ingestion Test (FIT)

**Figure 12:**
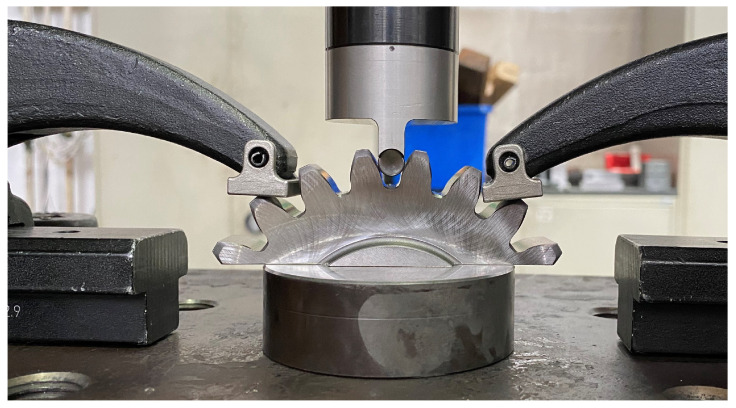
Experimental setup of the FIT.

To check the risk of fracture with the origin at the flank, cylindrical fragments are pressed quasi-statically into the tooth gap of the prepared planet gear (Figure 12). The numerical simulation proved that a fracture in the root occurs at the latest when the path of the fragment during ingestion is parallel to the tooth gap. As the stresses on the flank are also highest in this scenario, the risk of flank fracture is highest in this configuration. The fragments C3 and C4 are used. The fragment holder is designed to not come into contact with the tooth flanks during the test and not to undergo any deformation. The force of the occurring cracks is recorded and assigned to the “flank” or “root” fracture.

**Figure A1:**
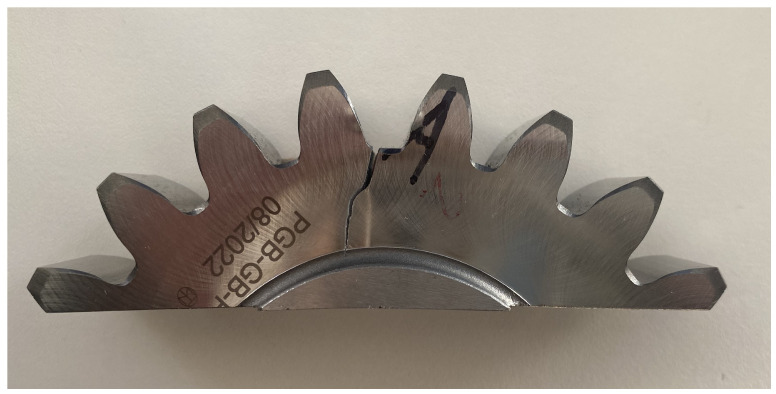
Crack propagation when indenting fragment C4 into the tooth gap (Test 2, front).

**Figure A2:**
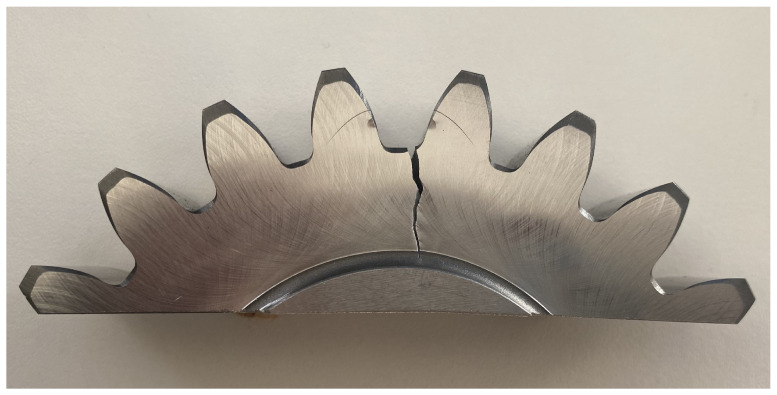
Crack propagation when indenting fragment C4 into the tooth gap (Test 2, back).

**Figure A3:**
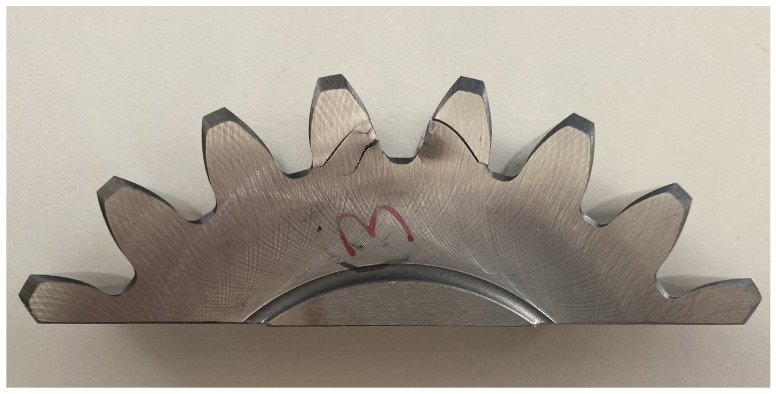
Crack propagation when indenting fragment C4 into the tooth gap (Test 3, front).

**Figure A4:**
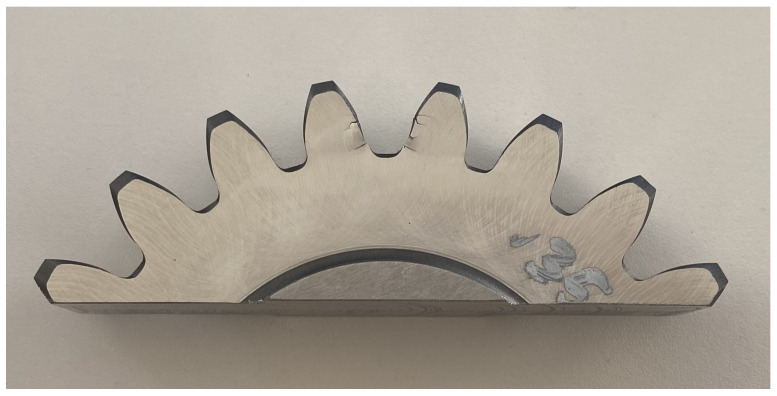
Crack propagation when indenting fragment C4 into the tooth gap (Test 3, back).

In the test series, the first and second tests show root fracture as the main failure, with a flank crack also visible on the facing edge. The main fracture in the third test originates at the flank with a secondary crack at the facing edge of the tooth root (Table 4). The crack characteristics of the flank fracture are comparable to those of a facing edge tooth flank fracture [21]. When fragment C3 is used, only root cracks occur. None of the cracks are symmetrical over the depth of the spur gear. Figures of the cracks are included in the Appendix A (Figure A1, Figure A2, Figure A3 and Figure A4).

## 3. Numerical Approach to Determine Constitutive Material Model

### 3.1. Determine Core Material Parameters

The nonlinear material properties of the core are determined by using the Johnson–Cook plasticity model since the test series will be extended to dynamic simulations in the future. The flow stress is calculated by:

(4) $$\sigma =\left(A+B\;\cdot \;\varepsilon^{n}\right)\;\cdot \;\left(1+C\;\cdot \;ln\left(\frac{\dot{\varepsilon }}{{\dot{\varepsilon }}_{0}}\right)\right)\;\cdot \;\left(1-{\left(\frac{T-T_{\mathrm{room}}}{T_{\mathrm{melt}}-T_{\mathrm{room}}}\right)}^{m}\right).$$

It involves the yield stress A, the hardening modulus B, the strain hardening exponent n, the strain rate sensitivity C, the reference plastic strain rate ${\dot{\varepsilon }}_{0}$, the thermal sensitivity m, and the melting and room temperature $T_{\mathrm{melt}}$ and $T_{\mathrm{room}}$. The strain rate and temperature term can be neglected for the present investigation since no significant temperature changes were observed in the quasi-static experiment. The material parameters, calculated based on the compression test, are shown in Table 5. As previously discussed in Section 2.1.2, the experimental results from the Vickers indenter test indicate a shift in the results for parameter A of the planet gear due to the influence of heat treatment on the core area.

### 3.2. Determine Case Material Parameters

Based on Moussa et al. [31], two layers in the case are necessary to represent the material behaviour at the hardened surface with a depth of 0.29 mm and the yield stress of 1962 MPa at the case and 315 MPa at the core with an error <1% compared to a 32 layer model. Since the surface layer is twice as thick for the ring gear and seven times as thick for the planetary and sun gear, the computing time is within an acceptable range and the error should be kept as low as possible, four layers are used. Each layer consists of three element rows. The calculated properties of the surface layer are determined based on a re-evaluation of the Vickers indentation tests using Equation (1). As mentioned in the introduction, there are different approaches for the constraint factor and the representative plastic strain. A comparison between the compression test and the determined hardness of the core shows that the values determined by Mata et al. [9] are suitable. The determined yield stresses are shown in Table 6.

### 3.3. Determine Homogenised Material Parameters

**Figure 13:**
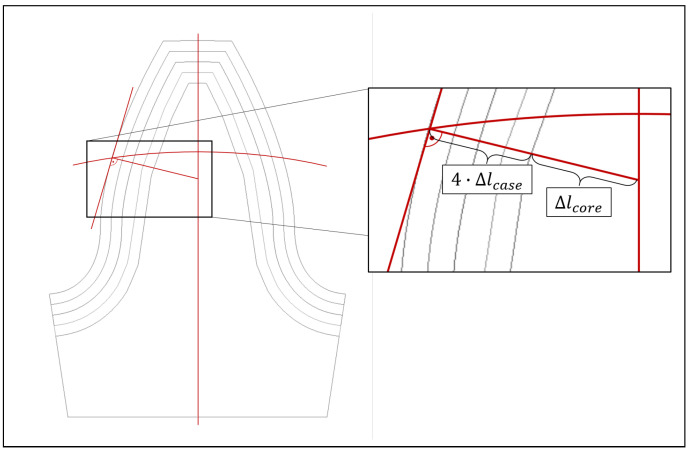
Homogenisation strategy for parameter A.

Due to the asymmetric load and the missing possibility of superposition, the entire planetary gearbox needs to be modelled. Taking the surface layer into account would lead to an excessively long calculation time. For this reason, a homogenised material model is used, and the number of elements is reduced to ten elements along the flank. To find the homogenised material properties based on the layered model, the rule of the mixture is modified based on the tooth geometry (Figure 13). For this purpose, the corresponding lengths are determined at the level of the pitch circle diameter, perpendicular to the tangent of the tooth flank. The homogenised yield stress $\sigma_{y,\mathrm{homogenized}}$ is thus given by

(5) $$\sigma_{y,\mathrm{homogenized}}=\frac{\sum_{i=1}^{4}\Delta l_{\mathrm{case}}\;\cdot \;\sigma_{y,i}+\Delta l_{\mathrm{core}}\;\cdot \;\sigma_{y,\mathrm{core}}}{4\;\cdot \;\Delta l_{\mathrm{case}}+\Delta l_{\mathrm{core}}}$$

where $\sigma_{y,i}$ is the yield stress in the corresponding surface layer i, $\sigma_{y,\mathrm{core}}$ is the yield stress of the core, $\Delta l_{\mathrm{core}}$ is the characteristic length of the core and $\Delta l_{\mathrm{case}}$ the thickness of one layer within the surface.

As the ring gear was designed with low radial stiffness to enable radial deflection, not only a deformation of the tooth itself but also a radial displacement of the ring rim must be taken into account. Simulations with a unit displacement to determine the stiffness of the ring rim have shown that, due to the thin surface layer, only the tooth in contact is influenced by the surface layer. The material properties of the gear rim itself comply with the core material.

## 4. Verification and Validation of the Constitutive Material Model

To verify the homogenised model, the comparability of the models is examined in terms of the plastic deformation, the risk of fracture and the initial location of the crack. The focus is on the root of the tooth, where the initial location of the crack is critical to its propagation and can make the difference between a catastrophic and benign outcome. To investigate the latter, the results of the pulsator test and the FIT are used. The mesh of the planet gear for the FIT and the pulsator test consists of 428.310 elements, whereby 56.160 elements are used for each surface layer. The length of the elements is around 0.2 mm. The fragments in the FIT, as well as the pulsator plates, are defined as rigid because no significant deformation was apparent on the through-hardened fragment. Quasi-static ingestion tests of a fragment into a scaled gearbox are used to validate the plastic behaviour of the homogenised model. The numerical simulation of this test cannot be solved in a reasonable time when using the layer model with fine meshing. Therefore, a coarse mesh with ten elements on the tooth flank is used. For numerical simulations without failure criteria, a quasi-static implicit calculation method is used. Otherwise, an explicit method is used due to instabilities and the resulting lack of convergence at the time of fracture.

### 4.1. Verify Homogenised Material Model Based on the Pulsator Test and FIT

**Figure 14:**
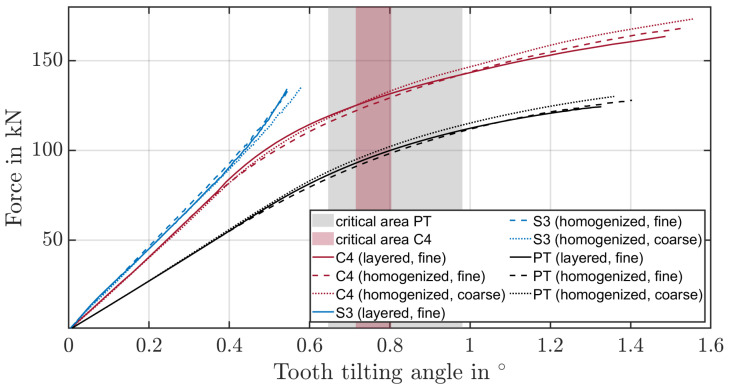
Tooth tilting angle to force obtained by the numerical simulation.

According to ISO 6336-3 [32], the tooth root stress depends on the nominal tangential force. This force generates a tilting deflection around the tooth root at the contact point. The tooth remains rigid and only the gear body deforms [33]. Since the contact point changes continuously during fragment ingestion, a tooth tilting angle is considered subsequently. Therefore, the required force-to-tooth tilting angle of the layered and homogenised models of the path-controlled pulsator test, as well as the FIT, were compared (Figure 14). The critical areas for the C4 fragment and the pulsator test are determined based on the maximum forces resulting in the experiments up to the point of fracture. The tooth tilting angle remains below the critical range for the spherical fragment S3. Here, the plastic deformation at the flank due to the point contact is the dominant effect. The relative error of the forces between the layered fine-mesh model and the homogenised fine- and coarse-mesh model at the mean fracture force is about 2% for the pulsator test and about 5% for the FIT. Overall, the material stiffness of the homogenised model is underestimated in both tests for small deformations and overestimated for large deformations that lead to plastic deformations in the core of the gear. Thus, the material properties for small deformations would have to be those of the surface layer and for large deformations those of the core material.

**Figure 15:**
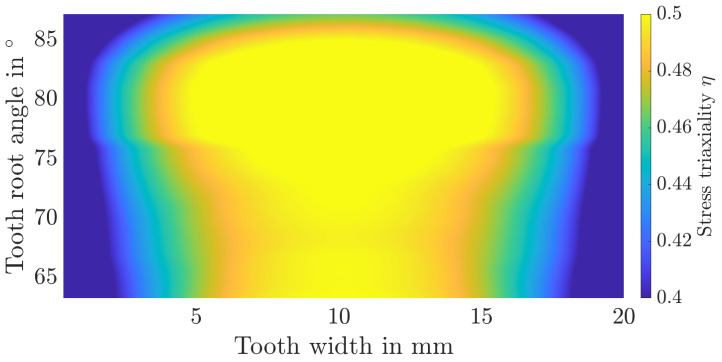
Distribution of the stress triaxiality over the tooth root area for the layered material model.

**Figure 16:**
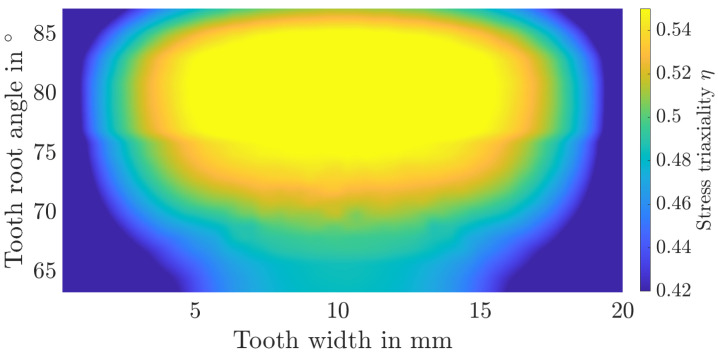
Distribution of the stress triaxiality over the tooth root area for the homogenised material model.

**Figure 17:**
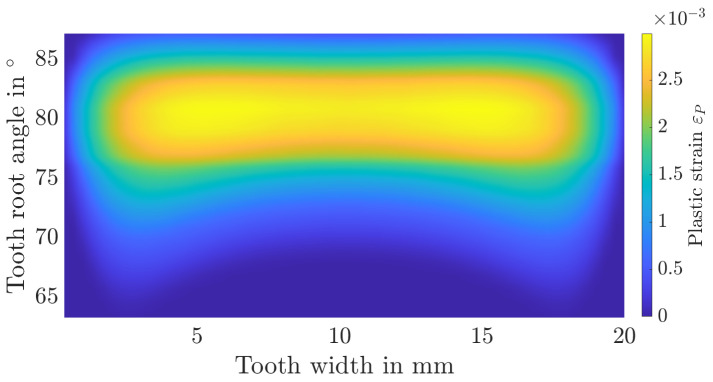
Distribution of the plastic strain over the tooth root area for the layered material model.

**Figure 18:**
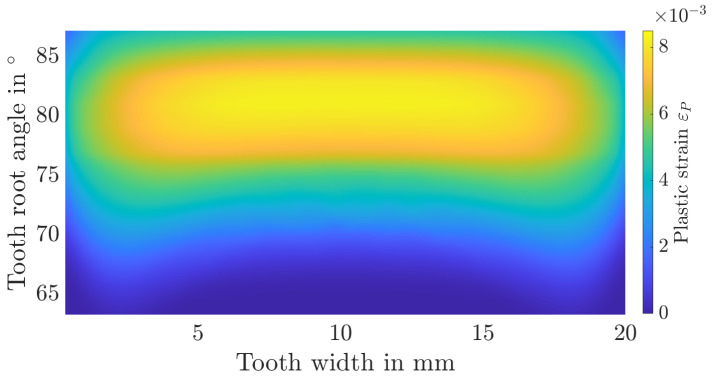
Distribution of the plastic strain over the tooth root area for the homogenised material model.

Additionally, the distribution of stress triaxialities as well as plastic strains over the tooth root is shown in Figure 15, Figure 16, Figure 17 and Figure 18. The tooth root angle is defined analogously to the initial crack location angle of Lewicki [34]. By homogenising the material and thus averaging the yield stresses, plastic deformation is higher overall. However, the local position of the maximum plastic strain remains comparable. The stress triaxiality increases slightly overall, the locale maximum remains comparable here as well. Within the numerical simulation, the maximum plastic strain or stress triaxiality, and therefore the initial crack position, is at a tooth root angle of 81.3°. In the experiments, the initial crack position was determined to be at an angle of approximately 82°. It can therefore be assumed that the initial crack location remains unaffected by the homogenisation.

### 4.2. Validate Homogenised Material Model Based on the Quasi-Static Ingestion Tests on the Scaled Gearbox

**Figure 19:**
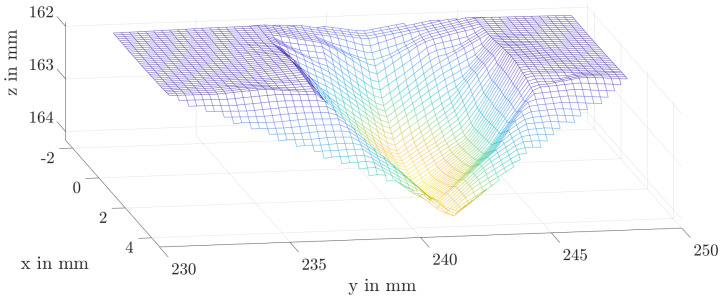
Plastic deformation after ingestion of a sphere (*D* = 8 mm) within the numerical simulation.

To validate the plastic deformation of the homogenised gear with only ten elements on the tooth flank of the planetary gear based on the experiments of the quasi-static ingestion tests, the penetration depth of the fragment into the tooth tip is compared. Beforehand, the influence of the number of elements over the chord length is evaluated. For this purpose, the number of elements is doubled, as well as quadrupled. Since the mesh density does not influence the penetration depth, the numerical simulations were then evaluated. To calculate the penetration depth, the edge of the tooth tip is taken as a reference. Figure 19 shows the results of the numerical simulation after the rollover of a sphere with a diameter of 8 mm.

For the experimental results, the penetration depth is measured by a laser sensor. Here, the sensor is moved along the tooth width. The results for the spherical fragments are shown in Table 7. When ingesting a spherical fragment with a diameter of 8 mm and an axial position relative to the gear width of 25% and 75%, the material fails at the tooth tip edge and begins to yield strongly due to softening and therefore it is not possible to measure any ingestion depth. According to Table 2, there are thus four tests with a spherical fragment in which the penetration depth can be compared. The average deviation of these depths is 1%. The consolidation behaviour of the implemented material thus agrees very well with the experimental results.

## 5. Determine the Fracture Locus of the Layered Material Model

Since cracks at the tooth root and the flank occurred during the FIT test, the fracture strain is to be defined using stress triaxiality. Since the spur gears of the scaled gearbox have a surface layer and therefore partly hardened and partly unhardened material, the fracture locus cannot be determined based on tensile, shear, and compression tests. Referring to the paper of Ghazali et al. [27], the applicability of a simple shift of the fracture locus, caused by the heat treatment of the material, is reviewed. As there were only plastic deformations and no initial cracks in the quasi-static ingestion tests on the scaled gearbox, only the pulsator test and the FIT can be used for determination and validation. However, an initial crack of the tooth flank was only present in the FIT. Therefore, the size of the shift is determined based on the results from the pulsator test. The results of the FIT are used as a basis for validation of this investigation. Since follow-up dynamic ingestion tests on the scaled gearbox will run under higher velocities, the Johnson–Cook failure model (Formula (3)) is used. For the quasi-static tests, both the velocity and temperature dependence can be neglected. To determine the fracture locus (Equation (3)), the three parameters D1, D2, and D3 must be determined. The gears failed in the pulsator test as well as in the FIT for high-stress triaxialities at low plastic strain values, while exhibiting ductile behaviour at lower or negative stress triaxialities. This needs to be taken into account when applying a failure model, which is commonly used for ductile materials.

### 5.1. Determination Based on Pulsator Test

**Figure 20:**
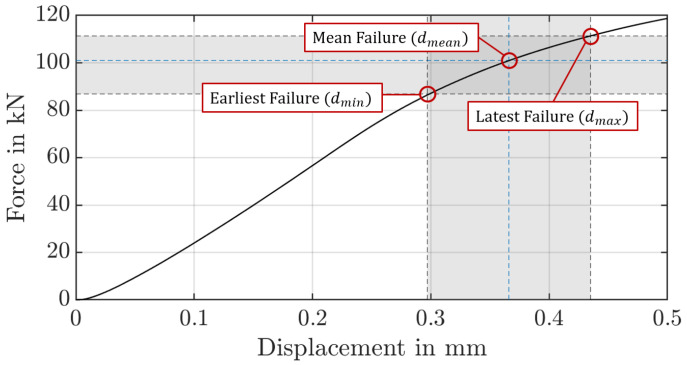
Displacement to force in the numerical simulation based on pulsator test.

**Figure 21:**
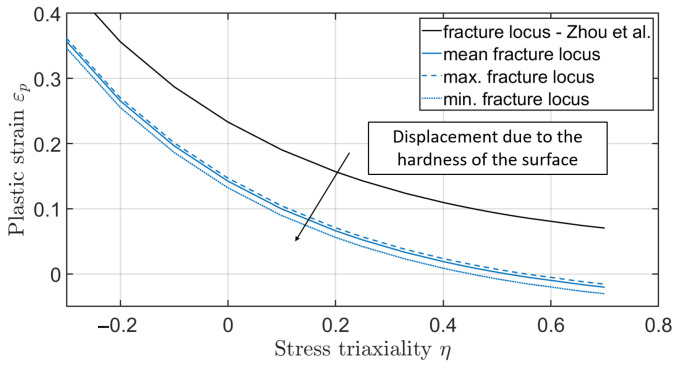
Determined shift of the known fracture locus based on pulsator test [35].

To determine the shift of the fracture locus due to the heat treatment, the fracture stain and the associated stress triaxiality value for the initial crack at the tooth root must first be determined. By comparing the measured forces in the pulsator experiment with the forces of the numerical model, these values can be determined from the numerical model. Based on the results of the numerical simulation a plane strain state can be assumed for the pulsator test [26]. To reduce the error due to the notch effect on the facing edge, the fracture strain is calculated based on the strain state in the centre of the gear. Figure 20 shows the force-displacement characteristic from the numerical simulation of the quasi-static pulsator test. The critical area is marked based on the forces at which the gear failed in the experiment and the corresponding displacements. Taking into account the scattering of the measured crack initiation times, the earliest crack in the experiment occurred at a displacement of $d_{min}=0.30\;mm$, and the latest crack was at $d_{max}=0.44\;mm$. Accordingly, the average indentation depth is $d_{mean}=0.37\;mm$. For later investigations, the critical range instead of the mean value is considered. In the numerical simulation, no plastic strains occur at the displacement $d_{min}$. The first plastic strain appears at a force of 90 kN and a displacement of $d_{min,FEM}=0.32\;mm$. The minimum fracture locus will be determined based on this penetration depth. Therefore, the minimum fracture strain is $\varepsilon_{min}=7\;\cdot \;10^{-8}$ with a stress triaxiality of η=0.48. For the averaged value of $F_{mean}=101\;kN$, the mean fracture strain is $\varepsilon_{mean}=8\;\cdot \;10^{-4}$ with a stress triaxiality of η=0.49. For the maximum force required to crack, the value of the maximum fracture strain is $\varepsilon_{max}=3\;\cdot \;10^{-3}$ with a stress triaxiality of η=0.52. By shifting the curve known from Zhou [35], the fracture locus for the layered material can thus be determined (Figure 21). Noticeably, the curve turns into negative fracture strains for stress triaxialities of around η=0.5 due to the axial displacement. The negative values result from the brittle material behaviour at high-stress triaxialities. This means any plastic deformation immediately leads to fracture.

### 5.2. Validation Based on FIT

The FIT strain state is used as a reference to validate the fracture locus determined from the pulsator test. Both root and flank fractures occurred during the test. Therefore, fracture strains are measured for different stress triaxiality states. First, the critical strain state at the root is compared with the determined fracture locus. It should be noted that although a plane strain state is also present for fragment C4, this assumption no longer holds for fragment C3. In this case, the length of the fragment is no longer equal to the width of the gear. Therefore, the fracture strains measured by FIT are expected to exceed the critical fracture strains determined from the pulsator test, as an axisymmetric stress state is present and, according to Xue et al. [26], the fracture locus is therefore above that of a plane strain state.

**Figure 22:**
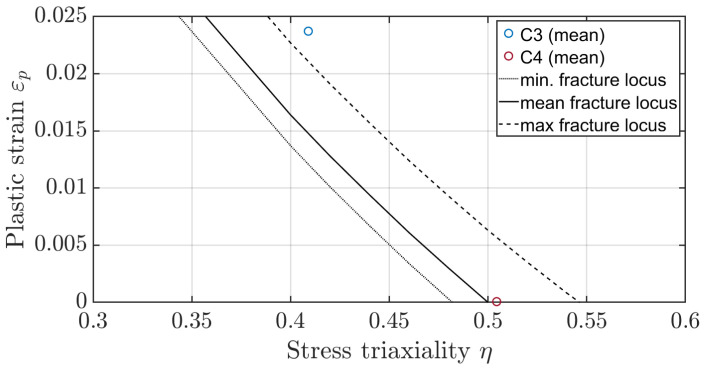
Comparison of the plastic strains at the tooth root for the fragments C3 and C4.

The averaged stress triaxiality to plastic strain values for the two cylindrical fragments C3 and C4 are shown in Figure 22. The results for fragment C4 agree very well with the fracture locus. However, the stress triaxiality is in the range where the plastic strain values become negative and thus fracture would occur with the onset of the first plastic deformations. The results for fragment C3 are, as expected, slightly above the critical region due to the axisymmetric stress state. Further testing would be required to determine this axisymmetric fracture curve.

**Figure 23:**
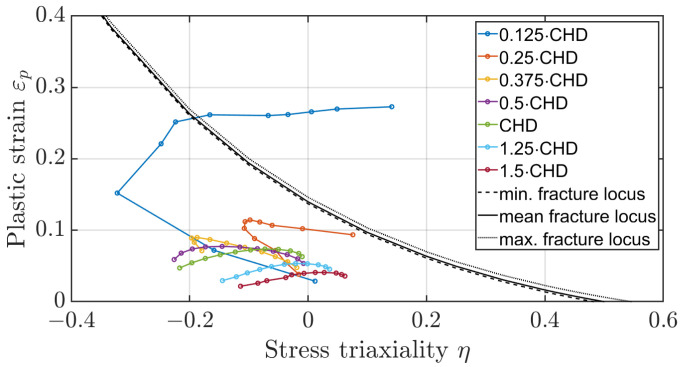
Plastic strain to stress triaxiality for layered flank elements at a distance of 0.5 mm of the edge at $d_{max}$.

**Figure 24:**
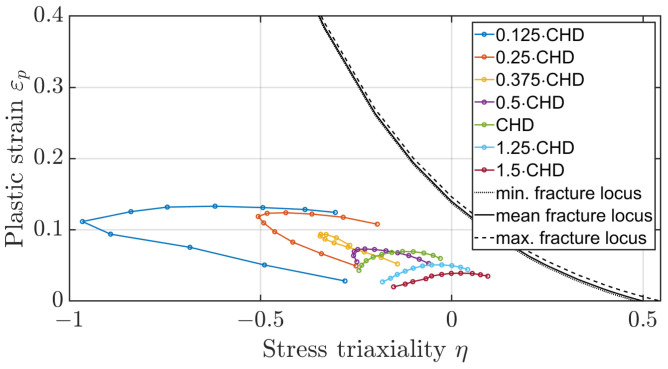
Plastic strain to stress triaxiality for layered flank elements at a distance of 1.5 mm of the edge at $d_{max}$.

The method of determining the fracture locus of a surface-hardened gear should also be evaluated based on the plastic strains occurring on the tooth flank. Figure 23 and Figure 24 show the mean stress triaxiality to plastic strain curves for the elements along the flank for different depths at $d_{max}$, referenced to the case hardening depth (CHD), once for a distance of 0.5 mm from the facing edge and once for a distance of 1.5 mm. It can be seen that the elements directly on the surface at the facing edge of the tooth would fail. According to the evaluations, the elements below the hardened layer are not critical, nor are the elements in the centre of the tooth. The experimental results of the third FIT, where the flank fracture is the main failure, appear to be caused by a defect in the material. When all other five FIT results are compared with the experiment, the crack initiation area is identical. The method of simple shift of the fracture locus as a function of heat treatment is suitable for a first estimation of the location of the main fracture. However, further experiments are necessary to investigate the effect of the third test in more detail.

## 6. Determine and Verify the Fracture Locus for the Homogenised Material Model

In addition to determining and validating the fracture locus for the layered model, the fracture locus for the homogenised material must also be determined and verified. The comparison of the stress triaxialities as well as the plastic strains across the tooth root for the layered and homogenised model is already done in Section 4.1 (Figure 15, Figure 16, Figure 17 and Figure 18).

**Figure 25:**
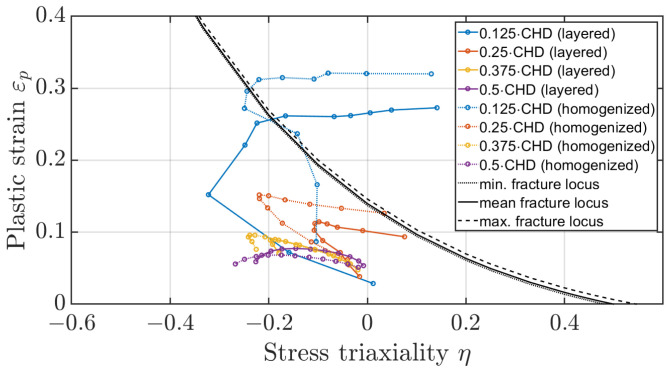
Comparison of plastic strain-to-stress triaxiality for layered and homogenised flank elements at a distance of 0.5 mm of the edge at $d_{max}$.

**Figure 26:**
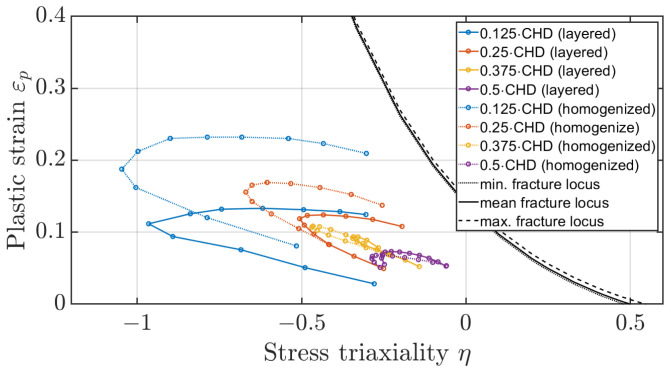
Comparison of plastic strain-to-stress triaxiality for layered and homogenised flank elements at a distance of 1.5 mm of the edge at $d_{max}$.

Taking a closer look at the plastic strains at the tooth flank at $d_{max}$ in the numerical model for a distance of 0.5 mm and 1.5 mm from the facing edge (Figure 25 and Figure 26), it can be seen that the magnitude of the plastic strains of the layer are 0.125·CHD and 0.25·CHD have shifted upwards in comparison to the layered model whereby the magnitude of the layers 0.375·CHD and 0.5·CHD are comparable. In addition, the elements with a distance of 0.25·CHD show a shift towards lower stress triaxiality values. In the homogenised model, the yield stress at the two outer layers is lower in comparison to the layered model and therefore plastic strains occur already at lower stresses. For the other two layers, the yield stress of the homogenised model deviates only about 6% and 11% from the yield stress of the associated layer of the layered model (Table 6).

**Figure 27:**
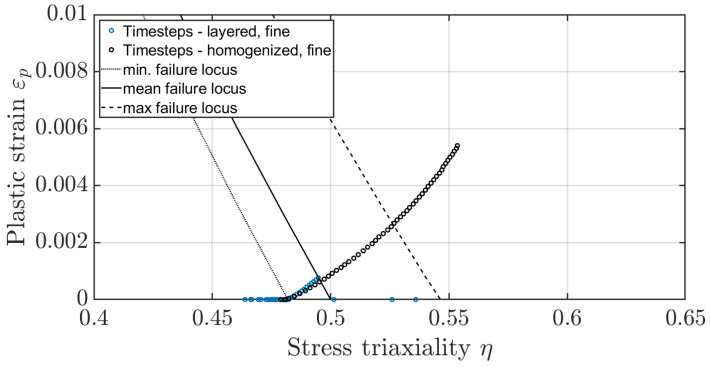
Comparison of the plastic strain-to-stress triaxiality of the layered and homogenised material model at the tooth root based on the pulsator test at $d_{mean}$.

**Figure 28:**
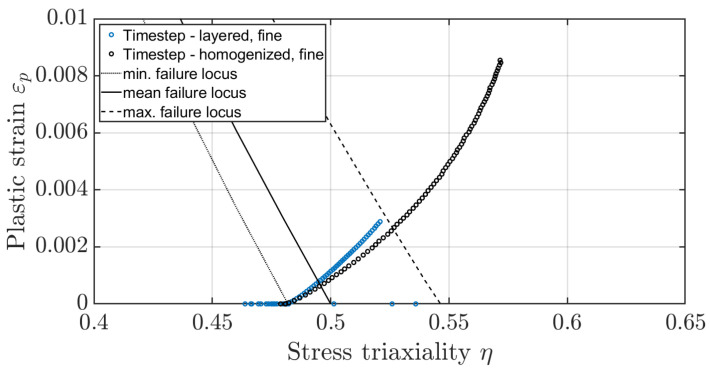
Comparison of the plastic strain-to-stress triaxiality of the layered and homogenised material model at the tooth root based on the pulsator test at $d_{max}$.

Comparing the averaged tooth root stress of the layered material model with the homogenised material model at $d_{mean}$ and $d_{max}$ shows that the progression of the plastic strain-to-stress triaxiality values with rising penetration depths is comparable across the indentation depth (Figure 27 and Figure 28), and only the rate of increase differs. One approach would therefore be to scale it based on Ghazali’s theory [27], since the plastic strains to stress triaxiality only differ in the rate but not in the path.

## 7. Summary

In this paper, the fracture locus and the resulting initial crack location were determined for the case of a fragment ingestion. Since such a load case has not yet been considered in the literature, it is necessary to investigate which modelling strategy is suitable for capturing the plastic deformations and the initial crack position with an adequate element size. Both a fine model including the surface layer and a homogenised material model, which is later suitable for enlarging the mesh elements, were determined. The fracture locus determined only applies to plane strain conditions. Furthermore, all tests were carried out quasi-statically, which is why the strain rate dependency was not considered. The analysed fragments were all of the same size as the tooth thickness. Residual stresses do not have to be taken into account in the numerical model. Therefore, parameters of the Johnson–Cook material model have been determined experimentally for the unhardened core based on compression tests and Vickers indenter tests for the nitrided ring gears (34CrAlNi7-10) and the case-hardened planet and sun gears (30CrNiMo8). The constitutive material model and the proposed homogenisation method were then validated experimentally using the quasi-static ingestion tests on the scaled gearbox and numerically verified based on FIT. Local plastic deformation on the tooth flank caused by the fragment and higher tooth tilting angles compared to general flank-to-flank contact will influence the initial crack position. As the initial crack position of the surface hardened and the homogenised model are comparable, homogenisation can also be used in such an application. To distinguish when the tooth flank rather than the root is the local origin of the damage, the feasibility of shifting the fracture locus as a function of hardness properties was investigated. Based on this fracture locus, it should later be possible to carry out a comprehensive numerical simulation to investigate which fragment shapes and which geometry parameters preferentially lead to catastrophic damage. So far, only spherical and cylindrical fragments with a diameter of 8 mm have been tested. To this aim, the parameters of the Johnson–Cook failure model for the case-hardened planetary gear were then determined using quasi-static pulsator tests. The parameters themselves and the curve-shifting approach were validated using FIT. Limitations were shown for a stress triaxiality above η=0.5. Here the curve became negative. However, since the fracture locus should only determine whether the damage occurs at the tooth flank or the root and stress triaxialities above η=0.5 are not of interest, the approach is sufficient for this application. Finally, the applicability of the approach depending on the homogenisation strategy was investigated. It was confirmed that the stress triaxiality to plastic strain values show a comparable course over the displacement and only the time of the first plastic deformation differs. Moving the curve dependent on the surface layer seems to be applicable. Possible limitations of this approach could not be identified during the investigations. This would require further tests with a greater variety of fragment shapes and gear shapes.

## Figures and Tables

**Table 1:** Gear basic geometry parameters.

Parameter	Value
Teeth number (Sun), $z_{S}$	24
Teeth number (Planet), $z_{P}$	21
Teeth number (Ring), $z_{R}$	66
Module, $m_{n}$	5 mm
Face width of gear, b	20 mm
Pressure angle, α	20°
Shifting coefficient, x	0.101

**Table 2:** Design of experiments (DOE) of the quasi-static tests.

Form	Sphere		Cylinder			
ID	S2	S3	C1	C2	C3	C4
Size	6	8	6 × 6	8 × 8	8 × 10	8 × 20
Position/gear width	25|50|75	25|50|75	25|50|75	25|50|75	25|50	50
Complete over-roll	x|x|x	x|x|x	x|x|x			

**Table 3:** Experimental results (quasi-static pulsator test).

Test-ID	max. Force [kN]
H33#02 V01	103.23
H33#02 V02	102.93
H33#02 V03	98.17
H33#02 V04	86.88
H33#02 V05	111.35
H34#02 V01	103.04

**Table 4:** Experimental results (FIT).

Test	Force—Initial Crack	Force—Secondary Crack	Fracture Location
1	128.6 kN	133.5 kN	root
2	125.9 kN	131.4 kN	root
3	106.0 kN	291.1 kN	flank
4	115.6 kN	123.4 kN	root
5	126.2 kN	149.2 kN	root
6	115.4 kN	132.5 kN	root

**Table 5:** Johnson–Cook parameters of 30CrNiMo8 and 34CrAlNi7-10.

Material	A	B	n
30CrNiMo8	322 MPa	912 MPa	0.3179
34CrAlNi7-10	729 MPa	565 MPa	0.3800

**Table 6:** Johnson–Cook parameter A of each layer of the surface and the core.

Material	Core	Layer 1	Layer 2	Layer 3	Layer 4
30CrNiMo8	1029 MPa	1160 MPa	1385 MPa	1600 MPa	1803 MPa
34CrAlNi7-10	741 MPa	960 MPa	1396 MPa	2087 MPa	3126 MPa

**Table 7:** Comparison: Ingestion depth of the fragment in FEM and experiment.

Config	Axial Position/Gear Width	$h_{\mathrm{ind}}$—FEM	$h_{\mathrm{ind}}$—Experiment
Sphere (d=6 mm)	50%	2.0	2.0
Sphere (d=6 mm)	25%	1.3	1.4
Sphere (d=6 mm)	75%	1.6	1.6
Sphere (d=8 mm)	50%	2.3	2.4

## Data Availability

The data presented in this study are available on request from the corresponding author.
